# Invasive paediatric *Elizabethkingia meningoseptica* infections are best treated with a combination of piperacillin/tazobactam and trimethoprim/sulfamethoxazole or fluoroquinolone

**DOI:** 10.1099/jmm.0.001021

**Published:** 2019-06-14

**Authors:** J. C. Chan, C. Y. Chong, K. C. Thoon, N. W. S. Tee, M. Maiwald, J. C. M. Lam, R. Bhattacharya, S. Chandran, C. F. Yung, N. W. H. Tan

**Affiliations:** ^1^ Department of Paediatrics, KK Women’s and Children’s Hospital, Singapore; ^2^ Yong Loo Lin School of Medicine, National University of Singapore, Singapore; ^3^ Department of Paediatrics, Infectious Disease Service, KK Women’s and Children’s Hospital, Singapore; ^4^ Duke-National University of Singapore Medical School, Singapore; ^5^ Lee Kong Chian School of Medicine, Nanyang Technological University, Singapore; ^6^ Department of Pathology and Laboratory Medicine, National University Hospital, Singapore; ^7^ Department of Microbiology, Yong Loo Lin School of Medicine, National University of Singapore, Singapore; ^8^ Department of Pathology and Laboratory Medicine, KK Women’s and Children’s Hospital, Singapore; ^9^ Department of Paediatric Subspecialties, Haematology/Oncology Service, KK Women’s and Children’s Hospital, Singapore; ^10^ Department of Neonatology, KK Women’s and Children’s Hospital, Singapore

**Keywords:** *Elizabethkingia meningoseptica*, hospital acquired infection, bacteraemia, meningitis, children

## Abstract

**Objectives:**

*
Elizabethkingia meningoseptica* is a multi-drug-resistant organism that is associated with high mortality and morbidity in newborn and immunocompromised patients. This study aimed to identify the best antimicrobial therapy for treating this infection.

**Methods:**

A retrospective descriptive study was conducted from 2010 to 2017 in a tertiary paediatric hospital in Singapore. Paediatric patients aged 0 to 18 years old with a positive culture for *E. meningoseptica* from any sterile site were identified from the hospital laboratory database. The data collected included clinical characteristics, antimicrobial susceptibility and treatment, and clinical outcomes.

**Results:**

Thirteen cases were identified in this study. Combination therapy with piperacillin/tazobactam and trimethoprim/sulfamethoxazole or a fluoroquinolone resulted in a cure rate of 81.8  %. The mortality rate was 15.4  % and neurological morbidity in patients with bacteraemia and meningitis remained high (75 %).

**Conclusions:**

Treatment with combination therapy of piperacillin/tazobactam and trimethoprim/sulfamethoxazole or a fluroquinolone was effective in this study, with low mortality rates being observed.

## Introduction

*Elizabethkingia meningoseptica* is a Gram-negative bacterium that is ubiquitous in soil and water, and also occurs in hospital settings [1]. It generally has low virulence, with a predilection for infecting newborns and immunocompromised hosts. It can also cause nosocomial outbreaks, especially in critical care and neonatal units, and these can be difficult to control [2]. It most commonly presents with meningitis and bacteraemia in neonates, but can also cause pneumonia and gastroenteritis, and, rarely, urinary tract infections, septic arthritis, ventriculitis, cellulitis and subdural haemorrhage in the paediatric population [3]. Endocarditis, abdominal infection, wound infection, sinusitis, epididymitis, dialysis-associated peritonitis and eye infections have also been reported in immunocompromised patients [4]. The reported risk factors for *E. meningoseptica* infection include prolonged hospital stay, the presence of comorbidities and the use of central venous catheters [5].

*E. meningoseptica* is resistant to many antimicrobial agents commonly used to treat Gram-negative infections, which often leads to ineffective treatment when the usual empirical antibiotics are chosen. The reported mortality rates are high (23–52 %) [6].

This retrospective study reviewed the clinical features of paediatric patients with *E. meningoseptica* sterile site infections at KK Women’s and Children’s Hospital over an 8-year period. Antimicrobial susceptibilities, treatment regimens and clinical outcomes were also evaluated.

## Methods

This was a retrospective 8-year descriptive study conducted between January 2010 and December 2017 at KK Women’s and Children’s Hospital, Singapore. Infants and children who were between 0 and 18 years old and had produced a positive culture for *E. meningoseptica* from any sterile site [blood, cerebrospinal fluid (CSF)] were included.

Cases were identified from the hospital laboratory database. Patients’ medical records were reviewed and the following information was extracted: age, gender, birth and neonatal history (if relevant), length of hospital stay prior to sterile site infection, highest level of care, clinical diagnosis, immunological status, presence of central venous access and removal (if applicable), antibiotic susceptibility, choice and duration of antibiotic treatment, clinical outcome, microbiological cure (defined as two successive negative blood cultures) and the ward or unit the patient was staying in at the time of laboratory confirmation.

### Microbiology

We used the Vitek^R^ mass spectrometry system (bioMérieux, Marcy-l’Etoile, France) or matrix-assisted laser desorption/ionization time-of-flight MS (bioMérieux SA, Marcy l’Etoile, France) to identify *E. meningoseptica *. Antimicrobial susceptibility test was performed using the CDS criteria for ‘* Chryseobacterium* sp.’ [7], and where no CDS criteria were available, we supplemented these with the Clinical and Laboratory Standards Institute (CLSI) criteria for ‘other non-* Enterobacteriaceae *’ [8] or the European Committee on Antimicrobial Susceptibility Testing (EUCAST) PK/PD non-species related breakpoints [9]. Cotrimoxazole was tested by disk testing, and the E-test strip (bioMérieux) was used for the other antimicrobials (piperacillin/tazobactam/ciprofloxacin/levofloxacin/moxifloxacin).

## Results

### Patients’ characteristics

During the 8-year study period, there were 13 patients with *E. meningoseptica* from blood (*n*=13) and CSF (*n*=4). Table 1 shows the clinical characteristics of these patients. Their ages ranged from 12 days to 9.5 years, with a median age of 2 years. Males constituted 46% of the patients and females constituted 54 % of the patients in this study. All patients were bacteraemic. In addition, four patients (three neonates) were also positive for *E. meningoseptica* in CSF and were diagnosed with meningitis.

**Table 1.** Clinical characteristics, antimicrobial treatment and clinical outcomes for patients with *E. meningoseptica* sterile site infections

Case	Age (years)	Sex	Location	Underlying condition	Date of isolation of blood culture	LOS prior to infection	CVL	Line removal (day of illness)	Meningitis	Antibiotic sensitivity	Empirical treatment	Treatment	Total duration of antibiotic treatment (days)	Time to first negative CS (days)	Neurological morbidity	Death
1	7	F	CICU	Acute lymphoblastic leukaemia	28 June 2011	4 days	Yes	No	No	TZP/SXT	CAZ/AMK/MNZ	TZP/SXT	14	3	No	No
2	9	F	CICU	Chronic granulocytic leukaemia	4 July 2011	5 months	Yes	Yes (1)	No	TZP, resistant to SXT, CIP	MEM/VAN	TZP/CIP	8 (till demise)	0	na	Yes
3†	8	F	Onco	Refractory acute myeloid leukaemia	21 July 2011	19 days	Yes	Yes (2)	No	TZP/SXT, resistant to CIP	MEM/AMK/VAN	SXT/CIP	21 days	1	na	Yes† *
4	0.5	F	NICU	Preterm 33 weeks, necrotizing enterocolitis	3 Mar 2012	6 months	Yes	No	No	TZP/SXT/CIP	MEM/AMK/VAN	TZP/SXT	14	5	Yes. Motor skills delay. Reduced academic performance	No
5	2	M	Onco	Pre-B acute lymphoblastic leukaemia	10 Apr 2012	1 day	Yes	Yes (8)	No	TZP/SXT, resistant to CIP	CAZ/GEN	TZP/SXT	14	44**	No	No
6	9.5	M	CICU	Pre-B acute lymphoblastic leukaemia	18 Nov 2012	2 months	Yes	No	No	TZP/SXT/MXF/LVX, resistant to CIP	MEM/VAN/CAZ	TZP/SXT	2 (till demise)	0	na	Yes
7	2	M	Ward	Haemophilia A with left subdural haematoma	23 Apr 2013	14 days	No	na	Yes	TZP/SXT/MXF/LVX, resistant to CIP	CRO	SXT/MXF, TZP^*a*^	42	2	Yes. Cerebral palsy, severe GDD	No
8	5	M	Ward	Extensive burns	15 June 2013	3 months	Yes	No	No	TZP/SXT/MXF/LVX, resistant to CIP	TZP	TZP/LVX, SXT^*b*^	14	9	No	No
9	2	M	Onco	Recurrent grade 4 pineoblastoma	30 June 2014	1 day	Yes	Yes (4)	No	TZP/SXT/MXF/LVX/CIP	TZP	TZP/CIP, SXT^*c*^	14	3	na	Yes*
10	20 days	F	NICU	Preterm 23 weeks	30 Sept 2014	20 days	Yes	Yes (14)	No	TZP/SXT/MXF/LVX/CIP	CXA/GEN	TZP/CIP, SXT^*d*^	14	8	Yes. Moderate GDD	No
11	13 days	F	NICU	Preterm 33 weeks, necrotizing enterocolitis	13 Apr 15	13 days	Yes	Yes (2)	Yes	TZP/RIF, resistant to SXT, CIP, MXF, LVX, MI	CXA/GEN/MNZ	TZP/RIF	21	9	No	No
12	30 days	F	NICU	Preterm 24 weeks	22 July 2015	30 days	Yes	Yes (2)	Yes	TZP/SXT/LVX/RIF	CXA/AMK	TZP/SXT	21	4	Yes. Moderate GDD	No
13	12 days	M	Ward	Term infant, left pelvi-ureteric junction obstruction	26 Apr 2016	1 day	No	na	Yes	TZP/SXT/MXF/LVX/CIP	AMP/GEN	TZP/SXT	21	6	Yes. Mild developmental delay. Hydrocephalus with VP shunt inserted	No

*a*, TZP discontinued due to allergic reaction; *b*, SXT discontinued due to allergic reaction; *c*, SXT discontinued due to plans for initiation of chemotherapy; *d*, SXT discontinued due to acute kidney injury.

* Patient died due to underlying oncological condition.

** Blood cultures were not performed on a regular basis due to palliative care.

† Patient 3 had two episodes of *E. meningoseptica* bacteraemia. During the first episode, she achieved a microbiological cure with intravenous trimethoprim/sulfamethoxazole and ciprofloxacin. Parents requested discharge against medical advice, and she was sent home on oral trimethoprim/sulfamethoxazole and ciprofloxacin to complete 21 days’ total treatment. She was readmitted 1 month later with a more resistant strain of *E. meningoseptica* bacteraemia [sensitive to TZP/CC/MI/MXF (intermediate) and resistant to SXT and CIP]. Although she managed to achieve a microbiological cure with a combination of piperacillin/tazobactam, clindamycin, minocycline and moxifloxacin, she succumbed to her underlying oncological condition.

AMK, amikacin; AMP, ampicillin; CAZ, ceftazidime; CC, clindamycin; CIP, ciprofloxacin; CRO, ceftriaxone; CVL, central venous line; CXA, cloxacillin; GDD, global developmental delay; GEN, gentamicin; LVX, levofloxacin; MEM, meropenem; MI, minocycline; MNZ, metronidazole; MXF, moxifloxacin; na, not applicable; RIF, rifampicin; SXT, trimethoprim/sulfamethoxazole; TZP, piperacillin- tazobactam; VAN, vancomycin; VP, ventriculoperitoneal shunt.

Seven patients (53.8 %) were immunocompromised, and of these six (46 %) had underlying malignancies and were being treated with chemotherapy, while one had severe third-degree burns. Four patients (30.7 %) were preterm infants, and two of these were extremely premature at 23 and 24 weeks, respectively. All immunocompromised patients and preterm infants had central venous catheters at the time of *E. meningoseptica* detection, and these were removed in 7/11 of the patients (63.6 %).

The average length of stay in hospital prior to developing *E. meningoseptica* bacteraemia was 45±57.3 days (range 1 to 180 days; median 19 days). Seven patients (53.8 %) acquired *E. meningoseptica* bacteraemia in the intensive care unit (ICU), while there were three patients (23 %) each from the oncology and general wards. All cases occurred sporadically, with no outbreaks or clusters of infection.

### Antimicrobial susceptibility

All isolates demonstrated 100 % susceptibility to piperacillin/tazobactam, and variable susceptibility to trimethoprim/sulfamethoxazole (11/14, 78.6 %) and fluoroquinolones (ciprofloxacin 4/12, 33.3 %; moxifloxacin 7/8, 87.5 %; levofloxacin 7/8, 87.5 %) (Table 1 and footnote).

Patient 3 had two episodes of *E. meningoseptica* bacteraemia. She was treated with intravenous trimethoprim/sulfamethoxazole and ciprofloxacin, and was able to achieve a microbiological cure. However, her parents requested that she be discharged against medical advice and she was sent home on oral trimethoprim/sulfamethoxazole and ciprofloxacin to complete 21 days’ total treatment. She was readmitted 1 month later with a more resistant strain of *E. meningoseptica* bacteraemia, which was resistant to both trimethoprim/sulfamethoxazole and ciprofloxacin. Although she eventually achieved a microbiological cure with a combination of piperacillin/tazobactam, clindamycin, minocycline and moxifloxacin, she succumbed to her underlying oncological condition.

### Therapeutic regimens and outcome

All patients were treated with combination antibiotics. Piperacillin/tazobactam was used in the treatment of all patients, except patient 3 during her first episode of *E. meningoseptica* bacteraemia. Piperacillin/tazobactam with trimethoprim/sulfamethoxazole was the most common antibiotic combination (*n*=6), followed by piperacillin/tazobactam with a fluroquinolone (*n*=4). The other antimicrobial agents used included minocycline, clindamycin and rifampicin. Patients 7 and 8 developed allergic reactions to piperacillin/tazobactam and trimethoprim/sulfamethoxazole, respectively, and were then changed to a fluoroquinolone. Trimethoprim/sulfamethoxazole was converted to ciprofloxacin in patient 9 in preparation for chemotherapy reinitiation to avoid the side effect of neutropaenia, while for patient 10 the switch was made in view of acute renal impairment.

A microbiological cure was achieved in 11 out of the 13 patients (84.6 %) in this case study.

### Mortality and morbidity

The rate for mortality attributable to *E. meningoseptica* bacteraemia was 15.4 % (2/13). Two patients achieved microbiological cure, but died from their underlying diagnosis (case 3 from refractory acute myeloid leukaemia and case 9 from recurrent grade 4 pineoblastoma). All neonates (4/4) in this case study survived.

Patients had been on follow-up from 2 to 5 years until the point of this study. Morbidity was high (75 %) amongst patients who presented with meningitis. Three out of four patients with meningitis were assessed to have developmental delay on follow-up. One patient had post-infectious hydrocephalus requiring ventriculo-peritoneal shunt insertion. Patient 7 developed quadriplegic cerebral palsy with severe disability, in addition to severe global developmental delay, although this likely resulted from the left subdural haematoma he sustained. Only one patient had appropriate growth and development when she was last reviewed at 2 years old.

Amongst patients who survived *E. meningoseptica* bacteraemia without meningitis (*n*=5), one patient had reduced academic performance, and another had moderate global developmental delay. Both patients were ex-premature infants, which could have contributed to the developmental delay. All patients with underlying malignancies who survived the infection (*n*=2) had no notable morbidity over a follow-up period of 5 years.

## Discussion

The main finding in our study is that combination therapy with piperacillin/tazobactam and trimethoprim/sulfaxazole or fluroquinolone seems to result in high rates of microbiological cure, with relatively low mortality.

There has been no consensus on the optimal antimicrobial therapy for *E. meningoseptica *. It has unusual antibiotic susceptibility profiles and is resistant to most antibiotics used to treat Gram-negative organisms, such as aminoglycosides, carbapenems and cephalosporins [5]. It has shown shown good susceptibility *in vitro* to inocycline, quinolones, trimethoprim/sulfamethoxazole, piperacillin/tazobactam and rifampin [1, 6]. Some anecdotal reports encourage the use of combination antibiotic therapy, especially in cases where monotherapy has failed [6]. Huang *et al*. [10] reported that monotherapy with fluoroquinolones had significantly better outcomes than monotherapy with non-fluoroquinolone antibiotics. In our study, all our patients were treated with combination antibiotics, with piperacillin/tazobactam as the backbone of the treatment. Piperacillin/tazobactam with trimethoprim/sulfamethoxazole was the most common combination used. All strains of *E. meningoseptica* in our study were susceptible to piperacillin/tazobactam, followed by trimethoprim/sulfamethoxazole (78.6 %) and fluoroquinolones (33.3–87.5 %). Amongst the fluoroquinolones, the highest sensitivities were observed with moxifloxacin and levofloxacin (87.5 %), while that for ciprofloxacin was only 33.3 %.

Case 3 was a patient whose infection initially responded very well to the combination of intravenous trimethoprim/sulfamethoxazole and ciprofloxacin, as was evident from the microbiological clearance after just 1 day of therapy. Unfortunately, she did not complete a course of intravenous antibiotics and was discharged on oral trimethoprim/sulfamethoxazole and ciprofloxacin due to her parents’ insistence. She returned to the hospital for *E. meningoseptica* bacteraemia again, which was resistant to trimethoprim/sulfamethxazole and ciprofloxacin the second time around. This suggests that oral antibiotics are insufficient for the treatment of *E. meningoseptica *. We postulate that the incomplete treatment of this infection resulted in the development of antimicrobial resistance, making it harder to treat subsequently.

Two patients were reported to have developed allergic reactions to piperacillin/tazobactam and trimethoprim/sulfamethoxazole, respectively. Trimethoprim/sulfamethoxazole was also changed to a fluoroquinolone in two other patients: a neonate with acute kidney injury and an oncological patient with planned chemotherapy. Trimethoprim/sulfamethoxazole have been associated with renal toxicity and cause bone marrow suppression, resulting in agranulocytosis, or aplastic anaemia, in previous studies. No other adverse effects from antimicrobial treatment were detected in this study.

Previous studies reported high mortality rates (33–52 %) [3, 6], with the highest mortality being observed among neonates [3]. In our study, the rate for mortality directly attributable to *E. meningoseptica* was much lower, at 15.4 %. All of the neonates in our study survived. Predictors of mortality include prematurity, immunocompromised state, delay in diagnosis, inappropriate antimicrobial treatment within 72 h of *E. meningoseptica* bacteraemia and central venous catheters [1, 6]. Our relatively low mortality rates may be due to early and appropriate combination antimicrobial therapy and newer antibiotic choices. Also, the first-line empirical antibiotic for children with febrile neutropaenia at our centre is piperacillin/tazobactam [11], which is usually effective in the treatment of *E. meningoseptica* infection.

Morbidity remained high in patients with meningitis; three out of four patients (75%) had hydrocephalus or neurological sequelae. Worldwide, 30 % of surviving children have developed hydrocephalus [3]. Other complications included developmental delays, seizures, deafness and growth retardation [3]. The morbidity from *E. meningoseptica* meningitis appears to be much higher than that for patients suffering from any other bacterial meningitis in our institution (23–29 %) [12].

In this study, the paediatric patients at greatest risk of *E. meningoseptica* sterile site infections were immunocompromised children with underlying malignancies (46 %), followed by neonates (less than 1 month of age; 30.7 %). Neonates were likely to present with meningitis together with bacteraemia, while older children were only positive for *E. meningoseptica* from blood samples. Other risk factors included prolonged hospital stay (mean length of hospitalization 45 days prior to diagnosis), admission to ICU and presence of a central venous catheter.

The number of reported cases of *E. meningoseptica* infection has steadily increased in recent years, with a worldwide geographical distribution [3]. Outbreaks of disease have been reported [13, 14], although the majority of cases occur sporadically. It is typically a nosocomial infection, and common sources include water supplies or medical equipment. Although previous studies have suggested that neonatal patients account for around three-quarters of the paediatric cases, they accounted for less than one-third of cases in our study, with the largest group being patients with haematological malignancies. Regular environmental surveillance, as well as intensified cleaning of medical equipment, have been effective in reducing *E. meningoseptica* outbreaks [6] and may be indicated in wards such as ICUs and oncology wards, where most at-risk patients are cared for.

We identified some limitations in our study. It was a retrospective descriptive study, with a small number of patients included, which is a reflection of the relatively low incidence of *E. meningoseptica* infections in our population. Further, antibiotic sensitivity testing for *E. meningoseptica* was only routinely performed for trimethoprim/sulfaxazole and piperacillin/tazobactam in our institute. The tests for other antibiotics such as fluoroquinolones were performed at the physician’s request, or if antimicrobial resistance to trimethoprim/sulfaxazole and/or piperacillin/tazobactam was detected. Future randomized controlled studies could be performed to identify the most effective combination antibiotic therapy in the treatment of *E. meningoseptica* sterile site infections.

### Conclusions

*E. meningoseptica* is an important cause of nosocomial infections, with high mortality and morbidity. Treatment with combination therapy of piperacillin/tazobactam and trimethoprim/sulfamethoxazole appeared to be effective in the majority of cases in our study, with comparatively low mortality rates being observed. Newer fluoroquinolones such as levofloxacin and moxifloxacin may be considered to be good alternatives, with high rates of sensitivity, and they could be effective for children who are unable to tolerate trimethoprim/sulfamethoxazole, with fewer adverse events being reported for them in our study. Future studies with randomized controlled trials of various combination therapies could be considered to evaluate efficacy.
